# Dramatic shift in the epidemiology of peptic ulcer in Japan: the impact of *Helicobacter pylori* eradication therapy

**DOI:** 10.1017/S095026882100265X

**Published:** 2021-12-06

**Authors:** K. D. Lee, T. Kayano, H. Nishiura

**Affiliations:** 1 Graduate School of Medicine, Hokkaido University, Kita 15 Jo Nishi 7 Chome, Kitaku, Sapporo 0608638, Japan; 2 Department of Infectious Diseases, Myeongji Hospital, Hanyang University Medical Center, Gyeonggi-do 10475, Seongdong-gu, Republic of Korea; 3 Kyoto University School of Public Health, Yoshidakonoecho, Sakyoku, Kyoto 606-8501, Japan

**Keywords:** Duodenal ulcer, epidemiology, *Helicobacter*, Japan, statistical model, stomach ulcer

## Abstract

*Helicobacter pylori* eradication therapy was included with insurance coverage from 1999 onwards in Japan, with the incidence of peptic ulcer expected to decrease as a consequence. This study investigated the temporal dynamics of peptic ulcer in Japan and identified underlying contributory factors using mathematical models. We investigated the seroprevalence of *H. pylori* and analysed a snapshot of peptic ulcer cases. Ten statistical models that incorporated important events – *H. pylori* infection, the cohort effect, eradication therapy and the natural trend for reduction – were fitted to the case data. The hazard of infection with *H. pylori* was extracted from published estimates. Models were compared using the Akaike information criterion (AIC), and factor contributions were quantified using the coefficient of determination. The best-fit model indicated that 88.1% of the observed snapshot of cases (AIC = 289.2) included the effects of (i) *H. pylori* infection, (ii) the cohort effect and (iii) eradication therapy, as explanatory variables, the contributions of which were 80.8%, 4.0% and 3.2%, respectively. Among inpatients, a simpler model with (i) *H. pylori* infection only was favoured (AIC = 107.7). The time-dependent epidemiological dynamics of peptic ulcers were captured and *H. pylori* infection and eradication therapy explained ⩾84% of the dramatic decline in peptic ulcer occurrence.

## Introduction

Peptic gastroduodenal ulcer is a defect in the gastrointestinal mucosa that reaches the muscularis mucosae [1]. Frequently, peptic ulcer occurs in the stomach or proximal duodenum [1–3]. Protective mechanisms exist in the gastrointestinal mucosa, including mucus and bicarbonate secretion; however, peptic ulcer develops when protection is weaker than the damaging factors, such as gastric acid and pepsin [1–3]. The spectrum of the disease varies from self-limiting pain or sensation to significant clinical complications, including bleeding and perforation [4]; emergency surgery is required for patients with severe perforation, which is responsible for the high mortality rate associated with peptic ulcers [5]. An important causative factor in the onset of peptic ulcer, *Helicobacter pylori* (a helix-shaped Gram-negative microaerophilic curved rod bacterium), has been identified in patients with chronic gastritis and gastric ulcer [6]. While the majority of people infected with *H. pylori* do not exhibit any clinical signs or symptoms, they may exhibit acute gastritis during the acute phase of infection. Moreover, chronic gastritis can lead to non-ulcer dyspepsia [7]. *H. pylori* infection is now regarded as the most important cause of peptic ulcer and gastric cancer [7, 8].

In Japan, the prevention of gastric cancer is a high priority because the mortality of gastric cancer has been consistently listed within the top three fatal cancers by body region [9]. Considering that *H. pylori* is a causative factor in the onset of peptic ulcer and gastric cancer, *H. pylori* eradication therapy is regarded as an important method for reducing the risk of these diseases [9]. Accordingly, the government of Japan approved national insurance coverage of eradication therapy as a treatment for *H. pylori*-positive gastroduodenal ulcers, beginning in the year 2000; in practice, coverage began in 1999 [9]. Insurance coverage was expanded in 2010 to include eradication therapy as a treatment for gastric mucosa-associated lymphoid tissue lymphoma, idiopathic thrombocytopenic purpura and post-endoscopic resection of early gastric cancer; in 2013, coverage was further expanded to cover eradication therapy as a treatment for *H. pylori*-positive gastritis [9, 10]. Because Japan has adopted universal health coverage, the impact of the insurance coverage policy is evident nationwide and anyone diagnosed with one of the abovementioned diseases can undergo eradication therapy with insurance coverage, such that they pay only 30% of the total cost [10].

An up-to-date global estimate of the prevalence and incidence (new cases) of peptic ulcer is 74.3 million people and 10.3 million person-years [11]. Major risk factors of peptic ulcer include psychological stress, *H. pylori* and the use of non-steroidal anti-inflammatory drugs (NSAIDs) [12]. The contribution of *H. pylori* to gastric ulcer has been reduced in Western countries, due to the reduced prevalence of *H. pylori*, and NSAIDs have become increasingly responsible for this disease [13]. In Japan, prior to the introduction of eradication therapy, the incidence of *H. pylori* declined dramatically over time, likely because the faecal-oral route of transmission became less common due to improved hygiene [8]. Therefore, the incidences of peptic ulcer and gastric cancer are both expected to decrease. However, the long-term dynamics of peptic ulcer have not yet been explored, especially since the introduction of eradication therapy [14–17].

The present study was performed to explore the temporal dynamics of peptic ulcer in Japan, with the aim of elucidating the underlying mechanism using parsimonious mathematical models. Multiple models were used to quantify the contribution of *H. pylori* eradication therapy to the reduced number of cases of peptic ulcer.

## Methods

### Epidemiological data

The present study focused on two pieces of information: the seroprevalence of infection with *H. pylori* in Japan and the snapshot case number of peptic ulcer in Japan. Regarding the seroprevalence of infection with *H. pylori*, a prior study by the authors estimated the time-dependent force of infection with *H. pylori* in Japan using systematically collected data ([18–24] as analysed in [25]). The year of survey, serological testing method, ages of subject and sample size are summarised in Supplementary Table S1. Briefly, survey years broadly ranged from 1974 to 2012, and the majority of surveys used serum IgG antibody, while only Akamatsu *et al*. used urine antibody among adolescents [19] and Okuda *et al*. used stool antigen and PCR [23]. Studies by Akamatsu *et al*. from 2007-9 [19] and Okuda *et al*. from 2010–11 [23] respectively focused on adolescents and school-age children, but other included studies examined the entire age spectrum involving both children and adults [18, 20–22, 24]. Despite such differences, our earlier study has shown that the observed antibody data over age and time, as extracted from all included studies, were well captured by a simple integral equation model and heterogeneity did not matter [25]. Using the collected dataset and estimating the hazard of infection [25], the best-fit model did not identify any age dependence in the hazard of infection and estimated that the hazard of infection decreased over time beginning in the year 1937; the exponential decay in the hazard was 0.047 per year [25]. We used these published estimates and adopted the resulting prevalence of infection with *H. pylori* as a function of time and age. There was no distinction in the force of infection by sex [25]; thus, we assumed that men and women shared an identical force of infection.

Regarding the snapshot case number of peptic ulcer, data were retrieved from a patient survey that is performed every 3 years by the Ministry of Health, Labour and Welfare in Japan (conducted in 1981, 1984, 1987, 1990, 1993, 1996, 1999, 2002, 2005, 2008, 2011 and 2014) [26]. This nationwide survey samples subject hospitals using a block randomisation method in which the blocking unit is the secondary medical region, defined as the geographic region in which healthcare facilities can offer a complete set of acute care services that require hospital admission (in 2019, *n* = 344 secondary medical regions in Japan). The principles of the survey method did not change over time. The survey collects information regarding patients' primary diagnoses, along with information regarding sex, date of birth, postal address, inpatient or outpatient status and treatment details; this data collection is performed to obtain an overview of the spectrum of diseases of both inpatients and outpatients in a single cross-sectional survey throughout Japan [27]. The survey is conducted on a single day in October of the survey year. The data were collected across age groups, regardless of a birth cohort. The total numbers of peptic ulcer cases derived from this survey, including both outpatients and inpatients, were 164 300 and 34 600, respectively, in 1984 and 2014. For this study, we retrieved the age-dependent number of peptic ulcer cases by sex, inpatient/outpatient information and survey year.

### Important events that lead to abrupt changes

In the present analysis, we captured the time-dependent case patterns of peptic ulcer by birth year cohort using mathematical models (see Statistical models); for this purpose, two important time events that likely affected the epidemiology of peptic ulcer are briefly described here. First, Japanese national health insurance began to cover the diagnosis and treatment of *H. pylori* infection for patients with peptic ulcer in 2000 (importantly, the implementation of coverage began in 1999) [28, 29]. The treatment guidelines for eradication therapy for *H. pylori* infection were also published during the same period [29]. The impact of change in 1999 was explored by statistical models 2-1, 2-2, 3-1 and 3-2. Second, prior to eradication therapy, cimetidine (an H2-receptor antagonist) was commercially introduced in Japan in 1982, and other H2-receptor antagonists (e.g. famotidine and ranitidine) were also introduced during the early 1980s [28]. This event was explored by our statistical models 3-1 and 3-2 in the present study. Accordingly, we set 1982 and 1999 as the years in which the epidemiological dynamics of peptic ulcer may have abruptly changed.

### Statistical model

Here, to explain the abrupt changes that are described in subsection ‘Important events that lead to abrupt changes’, we modelled the snapshot number of peptic ulcer and compared the model fit by including and excluding the following components: (i) seroprevalence of *H. pylori* infection; (ii) effect of birth year, proportional to *H. pylori* infection (hereafter referred to as the ‘cohort effect’); (iii) eradication therapy; and (iv) natural decline in the risk of infection. Let *i* be the index of birth year, such that *i* = {1925, 1935, 1945, 1955 and 1965}, and let *c*_i_(*t*) be the snapshot number of peptic ulcer in year *t*; then, the chronological age is given by *t*-*i*. Let *h*_i_(*t*) represent the seroprevalence of *H. pylori* infection in year *t*, which was previously parameterised in a published study [25]. Let *k* be a constant, expected value of the cases, such that the prototype model (M0) that does not include any event is as follows:1
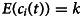
for *t* − *a* ≥ *i*. It should be noted that for the following models, we assume that the case number of peptic ulcer is dependent on the time series of *H. pylori* infection due to the established causal link to peptic ulcer, thus, all models other than (1) consider *H. pylori* seroprevalence. Assuming that *H. pylori* seroprevalence *h*_i_(*t*) is proportional to the risk of peptic ulcer, we have M1-1 as follows:2
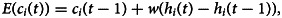
where *w* is the scaling factor of *H. pylori* infection. Extending M1-1 by considering the birth cohort effect in the element that is proportional to *H. pylori* infection, perhaps reflecting birth-cohort-specific susceptibility to *H. pylori* infection and associated complications, we have M1-2 as follows:3
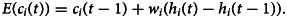


Additionally, considering an abrupt decline in the risk of peptic ulcer, possibly attained by eradication therapy, M2-1 was extended from M1-1 by a factor of (1 − ɛ), as follows:4
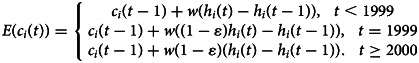


Accounting for the cohort effect in (3) onto M2-1, we have M2-2, as follows:5
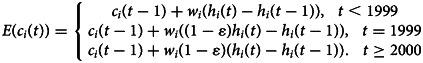
Adding the potential of a natural decline in peptic ulcer, which we assume was based on the introduction of H2-receptor antagonist therapy in 1982, expressed by the decay rate *δ* to the model in (4), we have M3-1, as follows:6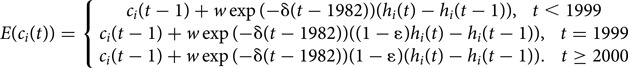 Accounting for cohort effect, we have M3-2, as follows:7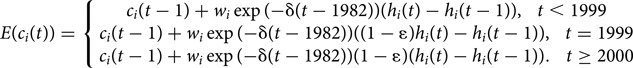 Assuming that the observed snapshot case number follows a Poisson distribution, unknown parameters (i.e. *w* (or *w*_i_), *ɛ* and *δ*) were estimated by means of maximum likelihood estimation using the likelihood function, as follows:8
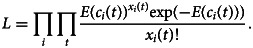
where *x*_i_(*t*) is the observed number of peptic ulcer cases in birth cohort *i* in year *t*. Fitted models were compared using the Akaike information criterion (AIC). Moreover, the coefficient of determination was computed to elucidate the percentage of observed time-dependent cases that could be described by the abovementioned simple components. Identifying the first and second best-fit models, we used the difference in the coefficient of determination to decompose the observed components into the abovementioned (i) through (iv), that is,
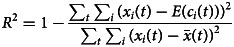
where 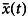 represents the mean of the observed data in year *t*.

Parametric bootstrapping was performed using the Hessian matrix H(***θ***). To do so, we have randomly drawn model parameters sampled from the normal distribution with the mean ***θ*** and standard deviation ***σ*** was obtained from the square root of diagonal elements of the inverse Hessian matrix. Then, for each set of parameters, we obtain a possible variation in epidemiological dynamics of peptic ulcer. By taking 2.5th and 97.5th percentile points of the simulated distributions, we obtain 95% confidence intervals of incidence.

Apart from the abovementioned time- and age-dependent models, it is critical to independently verify the presence of change in time trend in 1999 because of eradication therapy. To test our hypothesis that the frequency of peptic ulcer in Japan changed in 1999, we quantified the time trend using the following two models. First, if the frequency of peptic ulcers in Japan has been changing over time, independent of the event in 1999, the estimated case number *c* should be expressed as a simple linear function, such that9
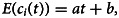
where *a* is the linear time-dependent trend and *b* is a constant parameter (intercept). Alternatively, if there was a changing point in 1999 due to the introduction of eradication therapy, the estimated frequency of peptic ulcers should be better described by10
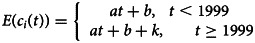
where *k* represents the change in the trend from 1999. Modelling *c* with those simple approaches, we performed a likelihood ratio test, comparing models (9) and (10), to identify a better-fit model for the empirically observed data.

### Ethical considerations

The present study used publicly available data [25, 27]. The datasets did not contain any information associated with individual identity; therefore, ethical approval was not required for this study.

## Results

**Fig. 1.**
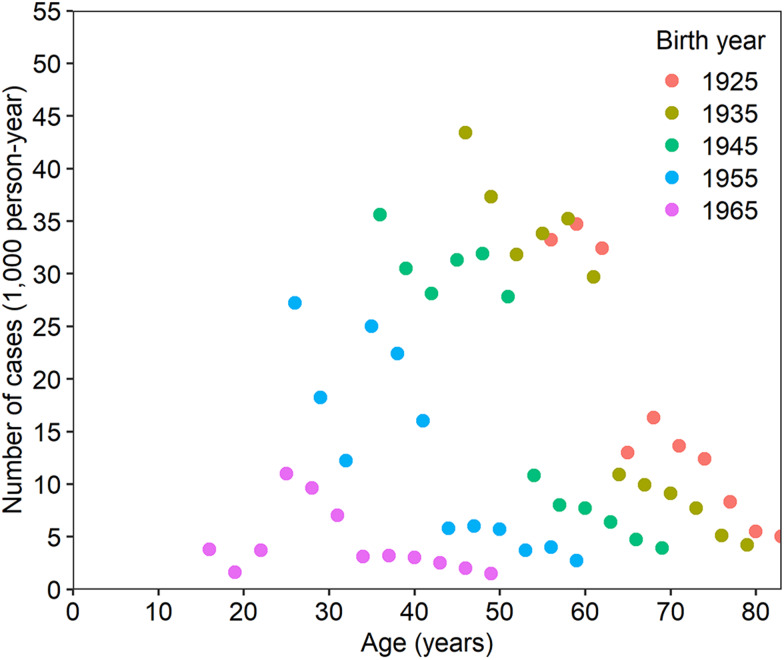
Snapshot number of peptic ulcer cases in Japan. The snapshot of peptic ulcer cases as a function of age and birth year. The dataset was collected from patient surveys of both men and women, including both inpatient and outpatient data. Each birth cohort is indicated by a unique symbol. The birth year plus age gives the year of observation.

As our descriptive datasets, Figure 1 shows the age-dependent snapshot case number of peptic ulcer for the total population, inclusive of both inpatients and outpatients, by birth cohort. Younger birth cohorts exhibited lower case values at an identical age, perhaps because younger cohorts followed a lower force of infection, as presented in our previous study [25]. Moreover, an abrupt decline in the snapshot case number was observed in each cohort when eradication therapy began to be covered by insurance in 1999.

Using the two models of the snapshot cases *c*_*i*_, as described by equations (9) and (10), we investigated whether the observed data were influenced by the event that occurred in 1999. Considering the result of the likelihood ratio test between the two models, the snapshot case numbers of peptic ulcer were better described in the model reflecting the change in 1999 than the simple linear function (see Supplementary Table S2 for model comparison and Table S3 for degrees of freedom). For all patients, including the male and female groups, there was a significant difference in the goodness-of-fit; that is, significance in accounting for the abrupt change from 1999, for four out of the five birth year cohorts available. This was the case for all birth cohorts among males.

Fitting seven different models with different complexities, we compared the AIC, the measure of goodness-of-fit, as shown in Table 1. The M2-2 model, which included (i) *H. pylori* seroprevalence, (ii) the cohort effect and (iii) eradication therapy, yielded the minimum AIC value and was thus regarded as the best model. The reduction resulting from *H. pylori* eradication therapy *ɛ* was estimated to be 0.93 (95% CI 0.71–1.11). For female patients only, the M2-1 model, including (i) *H. pylori* infection and (iii) eradication therapy, yielded the minimum AIC. When inpatient data were explored, the minimum AIC was obtained for M1-1, which included component (i) *H. pylori* infection only. When the total inpatient data were used with the M1-1 model, the scaling factor of *H. pylori w* was estimated to be 9.19 [95% confidence interval (CI) −31.29–47.87]. By contrast, the M3-2 model yielded the minimum AIC value for the total outpatient data, and this model included (i) *H. pylori* infection, (ii) the cohort effect, (iii) eradication therapy and (iv) an exponentially declining trend. M3-2 was the second-best model for the total population. It should be noted that all best-fit models included *H. pylori* infection as the explanatory variable.

**Table 1.** Comparison of models fitted to different datasets of peptic ulcer cases in Japan using the Akaike information criterion (AIC)

	Assumed model						
Dataset	M0	M1-1	M1-2	M2-1	M2-2	M3-1	M3-2
Total patients, total	809.4	320.6	315.4	308.7	289.2*	307.6	290.0
Total patients, male	666.9	269.8	265.3	263.4	250.0*	273.8	281.8
Total patients, female	325.3	199.0	205.5	195.6*	196.8	197.5	199.6
Inpatients, total	175.8	107.7*	113.0	108.2	113.9	109.5	115.4
Inpatients, male	1288.8	93.6*	99.3	94.8	100.6	96.3	102.3
Inpatients, female	76.1	52.9*	62.9	56.5	64.3	58.5	65.4
Outpatients, total	617.3	275.1	272.9	264.3	**245**.**8**	265.8	245.5*
Outpatients, male	500.0	227.8	226.9	200.1*	210.0	223.4	210.2
Outpatients, female	269.8	174.4	180.6	149.9*	172.5	172.8	175.8

AIC values are shown in each cell. M0, a single constant; M1-1, a model with *Helicobacter pylori* prevalence; M1-2, a model with *H. pylori* and the cohort effect on its weight; M2-1, a model with *H. pylori* and eradication therapy from 1999; M2-2, a model with *H. pylori* and the cohort effect on its weight, along with eradication therapy from 1999; M3-1, a model with *H. pylori* and eradication therapy from 1999, along with an exponential trend; M3-2, a model with *H. pylori* and the cohort effect on its weight, along with eradication therapy from 1999 and an exponential trend.

**Fig. 2.**
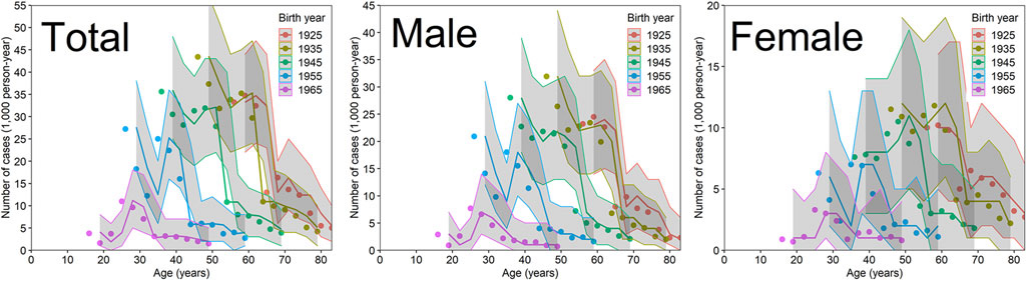
Comparisons of observed and predicted case numbers of peptic ulcer in Japan. Comparison of peptic ulcer cases against a model that includes *Helicobacter pylori* infection, eradication therapy and the cohort effect (M2-2). Coloured lines are overlaid with the corresponding birth cohort data (i.e. dots in the same colour). The total represents the sum of males and females. Grey shades represent the 95% confidence intervals of our model prediction using a parametric bootstrap method.

Figure 2 compares the observed and predicted case numbers of peptic ulcer for all inpatients and outpatients of both sexes. On the basis of visual assessment, M2-2, which included components (i), (ii) and (iii), well captured the observed relative prevalence by birth cohort and age. With the exception of the birth cohort born in 1925, models that account for the factor of reduction (1–*ɛ*) that measures the abrupt decline in case number attained by eradication therapy, coincided well with the observations.

Table 2 shows the estimated coefficient of determination, which is frequently denoted as *R*^2^, for each model and dataset; these coefficients can be interpreted as the proportions of variance in the observed peptic ulcer cases that were predicted using our parsimonious models. For datasets including both male and female populations, *R*^2^ was the highest with M3-2, showing the second-lowest AIC and including (i), (ii), (iii) and (iv); this yielded an *R*^2^ ranging from 0.84 to 0.88, indicating that our simple model explained approximately 85–90% of the observed pattern of peptic ulcer cases. For female populations, the highest *R*^2^ was obtained with M2-2.

**Table 2.** Adjusted coefficients of determination for different assumed models fitted to different datasets of peptic ulcer cases in Japan

	Assumed model						
Dataset	M0	M1-1	M1-2	M2-1	M2-2	M3-1	M3-2
Total patients, total	NC	0.81	0.79	0.84	0.88	0.85	0.88
Total patients, male	NC	0.82	0.81	0.85	0.89	0.82	0.82
Total patients, female	NC	0.77	0.75	0.81	0.86	0.81	0.85
Inpatients, total	NC	0.81	0.79	0.82	0.85	0.83	0.85
Inpatients, male	NC	0.80	0.79	0.81	0.84	0.82	0.84
Inpatients, female	NC	0.77	0.74	0.78	0.79	0.78	0.77
Outpatients, total	NC	0.76	0.74	0.80	0.86	0.80	0.87
Outpatients, male	NC	0.77	0.75	0.80	0.87	0.81	0.87
Outpatients, female	NC	0.72	0.70	0.78	0.84	0.79	0.83

NC, not calculable. M1-1, a model with *Helicobacter pylori* prevalence; M1-2, a model with *H. pylori* and the cohort effect on its weight; M2-1, a model with *H. pylori* and eradication therapy from 1999; M2-2, a model with *H. pylori* and the cohort effect on its weight, along with eradication therapy from 1999; M3-1, a model with *H. pylori* and eradication therapy from 1999, along with an exponential trend; M3-2, a model with *H. pylori* and the cohort effect on its weight, along with eradication therapy from 1999 and an exponential trend.

Table 3 decomposes the observed explainable proportions of each component from our model. For all patients (i.e. the sum of inpatients and outpatients), the contributions of (i), (ii) and (iii) were 80.8%, 4.0% and 3.2%, respectively (i.e. *H. pylori* and its eradication therapy combined were responsible for at least 84.0% of contributions). For all datasets, the contribution of eradication therapy was the greatest in terms of the cases of peptic ulcer, with the exception of *H. pylori* infection itself. Among inpatients, the impact of eradication therapy on the snapshot case number of peptic ulcer was approximately half that of its impact among outpatients or among the sum of inpatients and outpatients.

**Table 3.** Contribution of *Helicobacter pylori* infection, its eradication therapy and cohort and treatment effects on the observed time trend of peptic ulcer cases in Japan

	Contribution of functional terms (%)				
Dataset	*H. pylori*	Eradication therapy	Cohort effect	Exponential trend (treatment effect)	Total
Total patients, total	80.8	3.2	4.0	0.3	88.4
Total patients, male	82.3	2.6	3.8	−6.4	82.3
Total patients, female	76.7	4.6	4.3	0.4	85.2
Inpatients, total	80.7	1.7	2.5	0.2	85.0
Inpatients, male	80.2	1.3	2.7	0.2	84.4
Inpatients, female	76.7	1.6	0.0	−2.5	75.8
Outpatients, total	75.8	4.5	6.1	0.5	86.8
Outpatients, male	77.0	2.9	6.9	0.5	87.3
Outpatients, female	72.4	5.6	5.9	−0.8	83.0

The influence of (iii) eradication therapy was estimated by subtracting the *R*^2^ values of M1-1, a model with *H. pylori* and the cohort effect on its weight, from those of M2-1, a model with *H. pylori* and cohort effect on its weight, along with eradication therapy from 1999. Similarly, the contributions of (i) *H. pylori*, (ii) the cohort effect and (iv) the exponentially decreasing trend to the observed cases were estimated by considering the respective differences in *R*^2^ (i.e. M1-1 minus M0, M2-2 minus M2-1 and M3-2 minus M2-2). The best fit model M2-2 does not include the exponential effect, and the sum of the effects in M2-2 was 88.1%.

## Discussion

The present study describes the snapshot case number of peptic ulcer using parsimonious statistical models that highlighted the prevalence of *H. pylori* infection, as well as its eradication therapy and other important events that are presumably associated with the pathophysiological development of peptic ulcer. Models appeared to be useful for describing the relationship between *H. pylori* infection, its eradication therapy and other factors. Among the empirical data for peptic ulcer cases, the number decreased in younger birth cohorts and there was an abrupt decline in the snapshot case number when eradication therapy began to be covered by insurance in 1999, as detected by model comparisons using likelihood ratio testing. Seven different models were independently fitted to the observed data and the model that best described the observed time-dependent case number of peptic ulcer included (i) *H. pylori* seroprevalence, (ii) the cohort effect and (iii) eradication therapy as the explanatory variables.

As an important take-home message, *H. pylori* and its eradication therapy in combination contributed approximately 84% of the time series of peptic ulcer cases. The estimated reduction *ɛ* was estimated to be approximately 93% for the sum of inpatients and outpatients, and the population impact of eradication therapy (i.e. change in the trend) was explicitly identified by the likelihood-ratio test immediately after its introduction in 1999. In this regard, the impact of the changed insurance policy (added coverage of eradication therapy) on the cases of peptic ulcer was considered to be strongly positive. The remarkable reduction of peptic ulcer cases after the introduction of eradication therapy is believed to have reduced gastric cancer incidence [30], and this is the focus of our future investigations.

We also found that the best models among inpatients were different from those among others and a simpler model was favoured for *H. pylori* infection. Considering that patients with a severe clinical course rather than mild symptoms tended to be admitted into the hospital, inpatient data may represent the population who did not receive eradication therapy or ulcer medications. Low treatment effects, which accounted for half of the effects in the outpatient population, also reflect the fact that the inpatient population may not have received proper treatment in advance. These findings indicate that *H. pylori* infection alone is most responsible for the reduction in patients with more severe peptic ulcers, and that seroprevalence (reflecting the cumulative incidence) also affects the risk of severe disease.

Combining these two findings, it is plausible that future generations may not experience a substantial clinical burden from peptic ulcer. In particular, there are anticipated long-term effects of the natural decline in *H. pylori* infection and the indirect effect of eradication therapy. In addition to peptic ulcer cases, the incidence of gastric cancer should be carefully explored in future studies. By measuring the natural decline in the incidence of *H. pylori* infection, we determined that only a minor portion of the decline was induced by time-dependent progress in peptic ulcer treatment.

There were four important technical limitations in this study. First, the study relied on patient survey data, and the clinical validity of these patients' diagnoses is not fully established. It is likely that mild peptic ulcer patients included both acute and chronic gastritis patients to justify the use of H2-receptor antagonist therapy, proton pump inhibitor therapy and potentially even eradication therapy. Peptic ulcer cases may have been potentially overestimated. Second, because of the reliance on patient survey data, we were unable to determine whether recurrent cases were repeatedly included in calculations of the number of peptic ulcer cases. The cross-sectional data used in this study were based on hospital interviews, and more detailed explorations are needed in this regard to avoid possible overestimation of the impact of eradication therapy. Third, while we captured the overall observation patterns of the data, the calculated contribution is the proportion of variation described in the observed time series data regarding peptic ulcer cases; these were not attributable fractions or causal contributions of each component to the development of a peptic ulcer. The causative impact on the disease itself must be explored using a well-designed epidemiological study and individual datasets. Fourth, some important aspects of the data (e.g. a small reduction in the peptic ulcer cases in the old birth cohort at an early age) were not well explained by our models.

Despite limitations, we believe that our simple models successfully captured the time-dependent dynamics of peptic ulcer cases in Japan. Overall, there has been a dramatic decline in the snapshot number of peptic ulcer cases in Japan, and *H. pylori* infection and eradication therapy explained approximately 84% of this phenomenon.

## Data Availability

The data are only available in hardcopy written in Japanese, and the digitalised data are only available from the corresponding author on reasonable request.

## Supplementary material

For supplementary material accompanying this paper visit http://doi.org/10.1017/S095026882100265X. click here to view supplementary material
