# Supplementary material for: Dramatic shift in the epidemiology of peptic ulcer in Japan: the impact of Helicobacter pylori eradication therapy
Source: Epidemiol Infect. 2021 Dec 6;150:e4. doi: 10.1017/S095026882100265X (PMC8753483; doi:10.1017/S095026882100265X)
Supplement: Supplementary file 1 [file hygsup.zip › S095026882100265xsup001.docx]

Supplementary Table S1. The year of survey, serological testing method, ages of subject and sample size of seroepidemiological studies in Japan.

| Authors | Year of observation | Diagnostic testing method | Sample size | Ages of subject |
| --- | --- | --- | --- | --- |
| Fujisawa et al. | 1974  1984  1994 | Serum IgG | 349  324  342 | 0-70+  Table |
| Kumagai et al. | 1986  1994 | Serum IgG | 641  549 | 6-19 112  20-80 552 |
| Asaka et al. | 1991 | Serun IgG,  Urea breathing test | 426 | 0-6 75  High school and college 56  Health screening center 295 |
| Malaty et al | 1994 | Serum IgG | 480 | Adult 394  Children 86 |
| Akamatsu et al. | 2007  2008  2009 | Urine antibody | 409  370  445 | 16-17 |
| Hirayama et al. | 2008-2012 | Serum antibody | 21,144 | 30-79 |
| Okuda et al. | 2010  2011 | Stool antigen,  Stool real time polymerase chain reaction | 689  835 | 0-8  0-11 |
