# Supplementary material for: Dramatic shift in the epidemiology of peptic ulcer in Japan: the impact of Helicobacter pylori eradication therapy
Source: Epidemiol Infect. 2021 Dec 6;150:e4. doi: 10.1017/S095026882100265X (PMC8753483; doi:10.1017/S095026882100265X)
Supplement: Supplementary file 1 [file hygsup.zip › S095026882100265xsup002.docx]

Supplementary Table S2. Likelihood ratio test for model (10) to model (9) by birth cohort

| Year of birth | 1925 | | | 1935 | | | 1945 | | | 1955 | | | 1965 | | |
| --- | --- | --- | --- | --- | --- | --- | --- | --- | --- | --- | --- | --- | --- | --- | --- |
| cohort | Total | Male | Female | Total | Male | Female | Total | Male | Female | Total | Male | Female | Total | Male | Female |
| Log likelihood for model (9) | ˗12.53 | ˗3.17 | ˗29.98 | ˗18.51 | ˗14.45 | ˗10.40 | ˗15.84 | ˗12.43 | ˗10.15 | ˗14.92 | ˗11.88 | ˗9.02 | ˗11.51 | ˗9.51 | ˗6.78 |
| Log likelihood for model (10) | ˗12.01 | ˗10.45 | ˗8.38 | ˗13.36 | ˗11.01 | ˗9.89 | ˗11.99 | ˗10.80 | ˗9.25 | ˗12.41 | ˗10.26 | ˗8.58 | ˗9.44 | ˗8.02 | ˗6.22 |
| Test statistic | 2.37 | 24.43 | 99.49 | 23.67 | 15.81 | 2.36 | 17.73 | 7.50 | 4.17 | 11.56 | 7.43 | 2.04 | 9.51 | 6.86 | 2.60 |
| Degree of freedom | 1 | 1 | 1 | 1 | 1 | 1 | 1 | 1 | 1 | 1 | 1 | 1 | 1 | 1 | 1 |
| p-value | 0.12 | <0.001 | <0.001 | <0.001 | <0.001 | 0.12 | <0.001 | 0.01 | 0.04 | <0.001 | 0.01 | 0.15 | <0.001 | 0.01 | 0.11 |
