# Supplementary material for: Dramatic shift in the epidemiology of peptic ulcer in Japan: the impact of Helicobacter pylori eradication therapy
Source: Epidemiol Infect. 2021 Dec 6;150:e4. doi: 10.1017/S095026882100265X (PMC8753483; doi:10.1017/S095026882100265X)
Supplement: Supplementary file 1 [file hygsup.zip › S095026882100265xsup003.docx]

Supplementary Table S3. Degrees of freedom for different models employed to predict the peptic ulcer in Japan

| Subjects | M0 | M1-1 | M1-2 | M2-1 | M2-2 | M3-1 | M3-2 |
| --- | --- | --- | --- | --- | --- | --- | --- |
| Total | 52 | 52 | 48 | 51 | 47 | 50 | 46 |
| Male | 52 | 52 | 48 | 51 | 47 | 50 | 46 |
| Female | 52 | 52 | 48 | 51 | 47 | 50 | 46 |
